# Age-dependent survival in rapidly progressive glomerulonephritis: A nationwide questionnaire survey from children to the elderly

**DOI:** 10.1371/journal.pone.0236017

**Published:** 2020-07-13

**Authors:** Mayumi Takahashi-Kobayashi, Joichi Usui, Shuzo Kaneko, Hitoshi Sugiyama, Kosaku Nitta, Takashi Wada, Eri Muso, Yoshihiro Arimura, Hirofumi Makino, Seiichi Matsuo, Kunihiro Yamagata

**Affiliations:** 1 Medical Resident, University of Tsukuba Hospital, Tsukuba, Ibaraki, Japan; 2 Department of Nephrology, Faculty of Medicine, University of Tsukuba, Tsukuba, Ibaraki, Japan; 3 Department of Human Resource Development of Dialysis Therapy for Kidney Disease, Okayama University Graduate School of Medicine, Dentistry and Pharmaceutical Sciences, Okayama, Japan; 4 Department of Nephrology, Tokyo Women's Medical University, Tokyo, Japan; 5 Department of Nephrology and Laboratory Medicine, Kanazawa University, Kanazawa, Japan; 6 Department of Nephrology and dialysis, Kitano Hospital, Tazuke Kofukai Medical Research Institute, Osaka, Japan; 7 Department of Nephrology and Rheumatology, Kyorin University School of Medicine, Tokyo, Japan; 8 Kichijoji Asahi Hospital, Tokyo, Japan; 9 Okayama University, Okayama, Japan; 10 Nagoya University, Nagoya, Aichi, Japan; Universidade Estadual Paulista Julio de Mesquita Filho, BRAZIL

## Abstract

**Background:**

Rapidly progressive glomerulonephritis (RPGN) has been known to have a poor prognosis. Although evidence across adult RPGN cases has accumulated over many years, the number of case series in adolescents and young adults has been limited, requiring further studies.

**Methods:**

A total of 1,766 cases from 1989 to 2007 were included in this nationwide questionnaire survey, led by Intractable (former name, Progressive) Renal Diseases Research, Research on intractable disease, from the Ministry of Health, Labour and Welfare of Japan. To elucidate age-related differences in 2-year patient and renal survival rates, the cases were divided into the following four groups: children (0–18 years), young adults (19–39 years), the middle-aged (40–64 years), and the elderly (over 65 years).

**Results:**

Of the 1,766 total RPGN cases, antineutrophil cytoplasmic antibody (ANCA)-associated glomerulonephritis comprised 1,128 cases (63.9% of all RPGN cases), showing a tendency to increase with age. Two-year patient survival for RPGN was 93.9% among children, 92.6% in young adults, 83.2% in the middle-aged, and 68.8% in the elderly. The younger group (children plus young adults) showed a clearly higher survival rate compared to the older group (middle-aged plus elderly) (p<0.05). ANCA-associated glomerulonephritis also showed similar age-related results with all RPGN cases. The comparison of renal prognosis showed no statistically significant differences both in RPGN and in ANCA-associated GN.

**Conclusion:**

The present study described the age-dependent characteristics of the classification of RPGN, especially focusing on a better prognosis of the younger group in patient survival both in RPGN and in ANCA-associated GN.

## Introduction

Rapidly progressive glomerulonephritis (RPGN) is characterized by clinical features of progressive deterioration of renal function and glomerulonephritis, accompanied by active urinary sediment and crescent formation in histopathology [1]. Various etiologies lead to RPGN, including anti-glomerular basement membrane (GBM) antibody disease, immune complex deposition glomerulonephritis, and pauci-immune diseases associated with antineutrophil cytoplasmic antibody (ANCA) [2]. Efforts throughout the world have been made to grasp the whole picture of the disease; in Japan, a nationwide study conducted from 1989 to 2007 become the basis of the clinical guidelines in our country. Even though the prognosis has improved year by year, as shown in the mortality rate decrease from 38.7% to 18.0%, the prognosis in the elderly remains poor [3]. As the previous reports on this topic have mainly focused on adults, the studied cases in the relatively young population including children have been limited in number. In this context, age-based analysis, especially in pediatric and young adult cases, was determined to be the main purpose of this study.

## Materials and methods

### Study design

The study consisted of retrospectively collected data on patients with RPGN from 1989 to 2007 via a questionnaire survey sent to 351 institutes, including departments of nephrology, internal medicine, urology and pediatrics throughout the country (see S1 File) [3]. The total of 1,771 cases was registered from 178 different institutes. Cases were classified by age into the following four groups: children (0–18 years), young adults (19–39 years), the middle-aged (40–64 years), and the elderly (over 65 years). We conducted this survey with the permission of the medical ethics committee of the Graduate School of Comprehensive Human Sciences, University of Tsukuba, in accordance with the guidelines on epidemiological research from the Ministry of Health, Labour and Welfare of Japan. This study was conducted according to the Declaration of Helsinki. Informed consent of each individual to participate in this study was not required by the Institutional Review Board, because the study was a retrospective review of clinical records and pathological results only. Instead of informed consent of each individual, an announcement of this study was posted on each medical institution. The medical records of the patients were anonymized for personal information so that they cannot be identified.

### Clinical characteristics

Based on the questionnaire survey, all RPGN cases were categorized into 7 types of diagnosis and 27 classifications, in accordance with Glassock’s classification [4]. Of these 27 classifications of RPGN, pauci-immune-type crescentic GN (renal-limited vasculitis: RLV), microscopic polyangiitis (MPA) and granulomatosis with polyangiitis (GPA) were defined as antineutrophil cytoplasmic antibody (ANCA)-associated glomerulonephritis (GN). Clinical characteristics including age, gender, urinary protein, hematuria, serum creatinine, C-reactive protein (CRP), lung involvement, and treatment were also indicated by physicians on the questionnaire form. Estimated glomerular filtration rate (eGFR) was calculated using the equations applicable for Japanese children [5] and Japanese adults [6]. Lung involvement included chest X-ray abnormality, interstitial pneumonitis, pulmonary granuloma and pulmonary hemorrhage. Treatment was divided into glucocorticoid (GC), GC plus methylprednisolone (MP), GC plus cyclophosphamide (CY), GC plus MP plus CY, and no treatment.

### Statistical analyses

Two-year patient and renal survival rates were analyzed according to the four age categories (children, young adults, the middle-aged, and the elderly), and also the two categories, the younger group (children plus young adults), and the older group (middle-aged plus elderly) by Kaplan-Meier procedures and then statistically evaluated by log-rank tests using IBM SPSS Statistics for Windows, version 25.0 (IBM Corp., Armonk, N.Y., USA). P values less than 0.05 were considered to indicate significance.

## Results

### Diagnosis and classification

Of the total 1,771 cases, the 1,766 cases with age data were included in this study, comprising 42.0% RLV, followed by 19.4% MPA and 4.5% anti-GBM antibody-associated crescentic GN (Table 1). Within each diagnosis, RLV showed a greater population (81.1%) in primary crescentic GN, IgA nephropathy (58.1%) in primary GN with crescents, MPA (56.3%) in systemic disease-associated RPGN, poststreptococcal acute glomerulonephritis (26.3%) in infection-associated RPGN. ANCA-associated GN, including RLV, MPA and GPA accounted for 63.9% of RPGN (children 30.5%, young adults 36.8%, middle-aged 60.2%, elderly 73.2%). Anti-GBM antibody-associated crescentic GN and Goodpasture’s syndrome were observed in 6.0% of RPGN patients.

**Table 1:** Number of patients with RPGN, classified into four age categories.

Diagnosis	Classification	Age (years)									
		0–18		19–39		40–64		65-		Total	
		n	%	n	%	n	%	n	%	n	%
Primary crescentic GN	Anti-GBM antibody-associated crescentic GN	3	3.7	12	8.8	35	5.7	30	3.2	80	4.5
	Immune-complex-associated crescentic GN	2	2.4	5	3.7	12	1.9	16	1.7	35	2.0
	Renal-limited vasculitis	16	19.5	33	24.3	250	40.5	441	47.4	740	42.0
	Overlapped crescentic GN	3	3.7	0	0.0	11	1.8	17	1.8	31	1.8
	Unclassified primary crescentic GN	2	2.4	2	1.5	9	1.5	14	1.5	27	1.5
Primary GN with crescents	Membranoproliferative glomerulonephritis	2	2.4	1	0.7	1	0.2	11	1.2	15	0.8
	Membranous nephropathy	1	1.2	1	0.7	2	0.3	1	0.1	5	0.3
	IgA nephropathy	7	8.5	12	8.8	18	2.9	6	0.6	43	2.4
	Non-IgA mesangial proliferative GN	1	1.2	1	0.7	3	0.5	3	0.3	8	0.5
	Other primary GN	1	1.2	0	0.0	2	0.3	0	0.0	3	0.2
Systemic disease-associated	Goodpasture's syndrome	0	0.0	3	2.2	11	1.8	13	1.4	27	1.5
	Systemic lupus erythematosus	10	12.2	22	16.2	29	4.7	5	0.5	66	3.7
	Granulomatosis polyangiitis	4	4.9	8	5.9	23	3.7	11	1.2	46	2.6
	Microscopic polyangiitis	5	6.1	9	6.6	99	16.0	229	24.6	342	19.4
	Other necrotizing vasculitis	0	0.0	0	0.0	8	1.3	6	0.6	14	0.8
	IgA vasculitis	7	8.5	6	4.4	11	1.8	11	1.2	35	2.0
	Cryoglobulinemia	1	1.2	0	0.0	7	1.1	4	0.4	12	0.7
	Rheumatoid arthritis	0	0.0	1	0.7	12	1.9	11	1.2	24	1.4
	Malignant neoplasm	0	0.0	0	0.0	2	0.3	1	0.1	3	0.2
	Other systemic diseases	4	4.9	9	6.6	16	2.6	9	1.0	38	2.2
Infection-associated	Poststreptococcal acute glomerulonephritis	1	1.2	2	1.5	4	0.6	3	0.3	10	0.6
	Infective endocarditis, Shunt nephritis	1	1.2	2	1.5	1	0.2	1	0.1	5	0.3
	Sepsis, Abscess	0	0.0	0	0.0	0	0.0	2	0.2	2	0.1
	Hepatitis C virus	0	0.0	0	0.0	0	0.0	1	0.1	1	0.1
	Other infectious diseases	0	0.0	2	1.5	8	1.3	10	1.1	20	1.1
Drug-associated		0	0.0	1	0.7	5	0.8	4	0.4	10	0.6
Others		3	3.7	1	0.7	7	1.1	6	0.6	17	1.0
Unknown		8	9.8	3	2.2	32	5.2	64	6.9	107	6.1
Total		82	100.0	136	100.0	618	100.0	930	100.0	1766	100.0

According to age categories, in descending order of the proportion, children had RLV 19.5%, systemic lupus erythematosus 12.2%, IgA nephropathy and IgA vasculitis 8.5%, young adults had RLV 24.3%, systemic lupus erythematosus 16.2%, anti-GBM antibody-associated crescentic GN and IgA nephropathy 8.8%, middle-aged had RLV 40.5%, MPA 16.0%, anti-GBM antibody-associated crescentic GN 5.7%, and elderly had RLV 47.4%, MPA 24.6%, anti-GBM antibody-associated crescentic GN 3.2%. Thus, RLV comprised the highest percentage in each of the age groups (children 19.5%, young adults 24.3%, middle-aged 40.5%, elderly 47.4%), followed by systemic lupus erythematosus in children (12.2%) and young adults (16.2%), and MPA in the middle-aged (16.0%) and elderly (24.6%).

### Clinical characteristics of RPGN

Of the 1,766 of RPGN cases, there were 82 cases in children, 136 in young adults, 618 in the middle-aged, and 930 in the elderly (Table 2). The number of patients increased with age, and patients over 65 years accounted for more than a half of the total population (52.7%). The average age was 60.6 ± 17.6, with 832 males and 914 females. Most of the cases presented with proteinuria and hematuria (66.0%, 78.4%, respectively). The eGFR was lower and CRP was higher in the older patients than the younger patients with an average level of 17.2 mL/min/1.73m^2^ for eGFR, and 5.1 mg/dL for CRP, respectively. Especially, children had high eGFR (41.3±34.1 mL/min/1.73m^2^) and low CRP (1.8 ±2.9 mg/dL) level, compared to other age categories, young adults 25.3±23.4 mL/min/1.73m^2^, middle-aged 16.7±17.6 mL/min/1.73m^2^, elderly 14.5±13.3 mL/min/1.73m^2^ for eGFR, and young adults 3.4±6.3 mg/dL, middle-aged 4.8±6.5 mg/dL, elderly 5.9±6.5 mg/dL for CRP. The older group had more lung involvement than the younger group (children 15.9%, young adults 11.8%, middle-aged 36.2%, elderly 47.6%). The percentage of pulmonary hemorrhage was 8.5% in children, 4.4%in young adults, 10.7% in middle-aged, 11.5% in elderly. When focused on the group resulted in death within 2 years, lung involvement was seen in 20.0% of children, 30.0% of young adults, 32.0% of middle-aged, and 22.2% of elderly. As for the proportion of ANCA-associated GN, RLV accounted for 65.6%, followed by 30.3% of MPA and 4.1% of GPA, as more detailed information shown in Table 3.

**Table 2:** Clinical characteristics of RPGN, classified into four age categories.

			Age (years)				Total
			0–18	19–39	40–64	65-	
Number	(n)		82	136	618	930	1766
Age	(Average±SD years)		11.0±4.8	29.1±6.1	56.0±6.4	72.7±5.4	60.6±17.6
	(Median)		13	29	57	72	65
Gender	(Male / Female / Unknown)		38 / 44 / 0	56 / 80 / 0	289 / 322 / 7	449 / 468 / 13	832 / 914 / 20
Urinary protein	(n, %)	Positive	59, 71.9%	102, 75.0%	450, 72.8%	555, 59.7%	1166, 66.0%
		Negative	8, 9.8%	23, 16.9%	104, 16.8%	204, 21.9%	339, 19.2%
		Missing	15, 18.3%	11, 8.1%	64, 10.4%	171, 18.4%	261, 14.8%
Hematuria	(n, %)	Positive	68, 82.9%	120, 88.2%	495, 80.1%	701, 75.4%	1384, 78.4%
		Negative	6, 7.3%	11, 8.1%	56, 9.1%	97, 10.4%	170, 9.6%
		Missing	8, 9.8%	5, 3.7%	67, 10.8%	132, 14.2%	212, 12.0%
Serum creatinine	(Average±SD mg/dL)		2.6±2.2	4.0±3.8	4.8±3.4	4.9±3.2	4.7±3.3
eGFR	(Average±SD mL/min/1.73m^2^)		41.3±34.1	25.3±23.4	16.7±17.6	14.5±13.3	17.2±18.0
CRP	(Average±SD mg/dL)		1.8±2.9	3.4±6.3	4.8±6.5	5.9±6.5	5.1±6.5
Lung involvement	(n, %)		13, 15.9%	16, 11.8%	224, 36.2%	443, 47.6%	696, 39.4%
—Pulmonary hemorrhage	(n, %)		7, 8.5%	6, 4.4%	66, 10.7%	107, 11.5%	186, 10.5%
ANCA-associated GN	(n, %)	RLV	16, 64.0%	33, 66.0%	250, 67.2%	441, 64.8%	740, 65.6%
		MPA	5, 20.0%	9, 18.0%	99, 26.6%	229, 33.6%	342, 30.3%
		GPA	4, 16.0%	8, 16.0%	23, 6.2%	11, 1.6%	46, 4.1%

**Table 3:** Clinical characteristics of ANCA-associated GN, classified into four age categories.

			Age (years)				Total
			0–18	19–39	40–64	65-	
Number	(n)		25	50	372	681	1128
Age	(Average±SD years)		11.3±5.3	29.6±6.2	57.1±5.7	72.9±5.5	64.4±14.4
	(Median)		13	30	58	72	67
Gender	(Male / Female / Unknown)		12 / 13 / 0	16 / 34 / 0	184 / 183 / 5	322 / 350 / 9	534 / 580 / 14
Urinary protein	(n, %)	Positive	21, 84.0%	37, 74.0%	277, 74.5%	406, 59.6%	741, 65.7%
		Negative	2, 8.0%	7, 14.0%	57, 15.3%	161, 23.6%	227, 20.1%
		Missing	2, 8.0%	6, 12.0%	38, 10.2%	114, 16.7%	160, 14.2%
Hematuria	(n, %)	Positive	23, 92.0%	45, 90.0%	305, 82.0%	533, 78.3%	906, 80.3%
		Negative	1, 4.0%	3, 6.0%	29, 7.8%	65, 9.5%	98, 8.7%
		Missing	1, 4.0%	2, 4.0%	38, 10.2%	83, 12.2%	124, 11.0%
Serum creatinine	(Average±SD mg/dL)		2.9±1.9	3.7±2.4	4.7±3.1	4.7±2.9	4.6±2.9
eGFR	(Average±SD mL/min/1.73m^2^)		41.2±34.2	21.2±17.5	16.1±14.2	14.7±13.3	16.0±14.9
CRP	(Average±SD mg/dL)		2.9±3.8	2.7±4.1	4.9±6.3	6.0±6.3	5.4±6.2
Lung involvement	(n, %)		9, 36.0%	7, 14.0%	166, 44.6%	356, 52.3%	538, 47.7%
—Pulmonary hemorrhage	(n, %)		6, 24.0%	3, 6.0%	45, 12.1%	88, 12.9%	142, 12.6%
ANCA-associated GN	(n, %)	RLV	16, 64.0%	33, 66.0%	250, 67.2%	441, 64.8%	740, 65.6%
		MPA	5, 20.0%	9, 18.0%	99, 26.6%	229, 33.6%	342, 30.3%
		GPA	4, 16.0%	8, 16.0%	23, 6.2%	11, 1.6%	46, 4.1%
ANCA positivity	(n, %)	MPO-ANCA	17, 68.0%	29, 58.0%	275, 73.9%	602, 88.4%	923, 81.8%
		PR-3 ANCA	3, 12.0%	8, 16.0%	21, 5.6%	16, 2.3%	48, 4.3%
		Both positive	0, 0.0%	4, 8.0%	24, 6.5%	0, 0.0%	28, 2.5%
		Both negative	5, 20.0%	8, 16.0%	35, 9.4%	48, 7.0%	96, 8.5%
		Missing	0, 0.0%	1, 2.0%	17, 4.6%	15, 2.2%	33, 2.9%
Treatment	(n, %)	GC	4, 16.0%	6, 12.0%	50, 13.4%	152, 22.3%	212, 18.8%
		GC+MP	7, 28.0%	26, 52.0%	174, 46.8%	310, 45.5%	517, 45.8%
		GC+CY	5, 20.0%	4, 8.0%	32, 8.6%	51, 7.5%	92, 8.2%
		GC+MP+CY	6, 24.0%	3, 6.0%	61, 16.4%	83, 12.2%	153, 13.6%
		No treatment	1, 4.0%	1, 2.0%	16, 4.3%	37, 5.4%	55, 4.9%
		Missing	2, 8.0%	10, 20.0%	39, 10.5%	48, 7.0%	99, 8.8%

### Clinical characteristics of ANCA-associated GN

Focusing on ANCA-associated GN, as represented by RLV, MPA and GPA, the total number of patients was narrowed down to 1,128 (accounted for 63.9% of RPGN, Table 1), including 25 cases in children, 50 in young adults, 372 in the middle-aged, and 681 in the elderly (Table 3). Age, eGFR, and CRP of ANCA-associated GN showed tendencies similar to those in the total RPGN group. Lung involvement was seen in 47.7% in total, children 36.0%, young adults 14.0%, middle-aged 44.6%, elderly 52.3%, respectively. The percentage of pulmonary hemorrhage was the highest in children (24.0%), followed by elderly (12.9%), middle-aged (12.1%), young adults (6.0%). As for the proportion of ANCA-associated GN, the older patients showed a greater proportion of MPA (children 20.0%, young adults 18.0%, middle-aged 26.6% and elderly 33.6%), whereas GPA was more heavily weighted towards the younger groups (children 16.0%, young adults 16.0%, middle-aged 6.2%, elderly 1.6%). Almost half of the ANCA-associated GN patients were treated with a combination of glucocorticoids (GC) and methylprednisolone (MP), comprising the largest proportion in any age group (total 45.8%, children 28.0%, young adults 52.0%, middle-aged 46.8%, elderly 45.5%). Treatment with cyclophosphamide (CY), including GC plus CY or GC plus MP plus CY, was administered in 44.0% of children, 14.0% of young adults, 25.0% of the middle-aged and 19.7% of the elderly. Thus, the use of CY was more frequent in children compared to other age categories.

### Patient and renal survival

**Fig 1:**
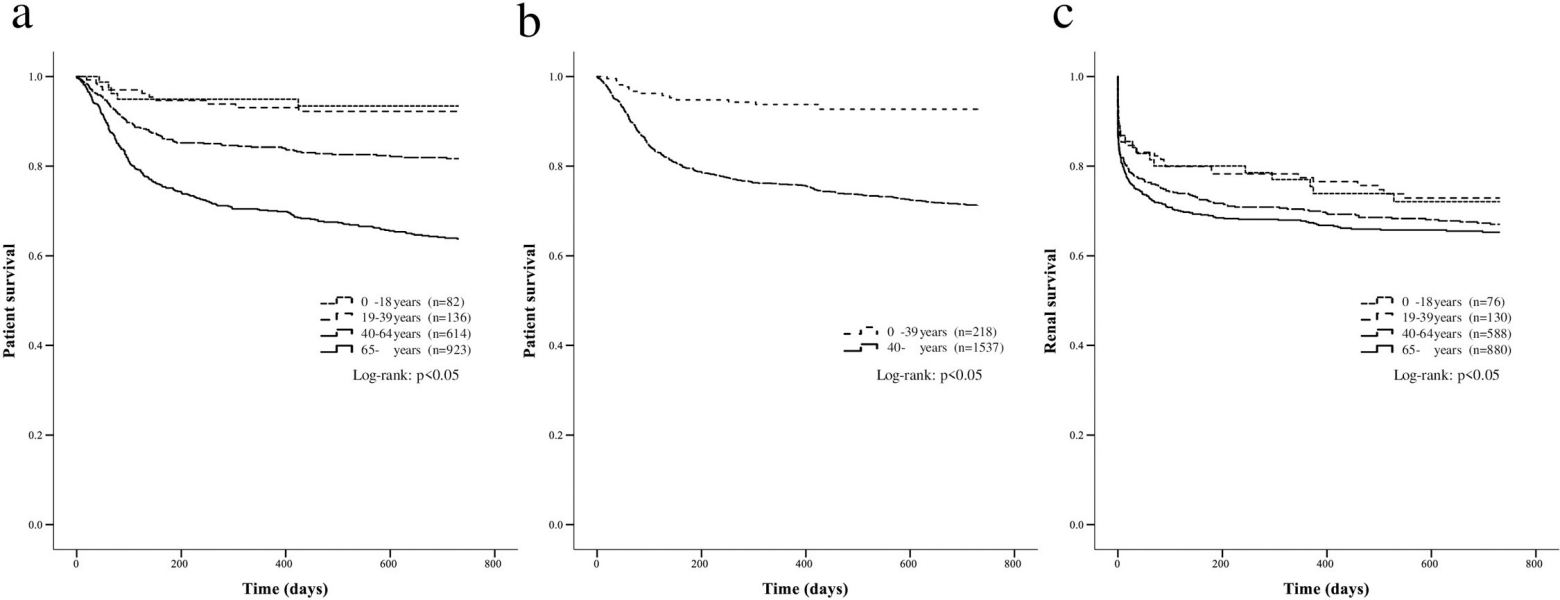
The patient and renal survival in patients with RPGN. The comparison of patient survival in patients with RPGN among four age groups (A) and two age groups (B), and the renal survival in patients with RPGN among four age groups (C) (A, B: n = 1,755, C: n = 1,674).

During the 2-year follow-up period, the survival rate of RPGN was significantly different among four age groups, 93.9% in children, 92.6% in young adults, 83.2% in the middle-aged, and 68.8% in the elderly (p <0.05) (Fig 1A). Also, when comparing the younger group (children plus young adults) with the older group (middle-aged plus elderly), the survival rate of the younger group was 93.1%, representing significantly better prognosis compared to 74.6% in the older group (p <0.05) (Fig 1B). Among these RPGN patients, there were no statistically significant differences in the renal survival rate of this period, 73.7% in children, 73.8% in young adults, 69.0% in the middle-aged, and 68.1% in the elderly (p = 0.15) (Fig 1C).

**Fig 2:**
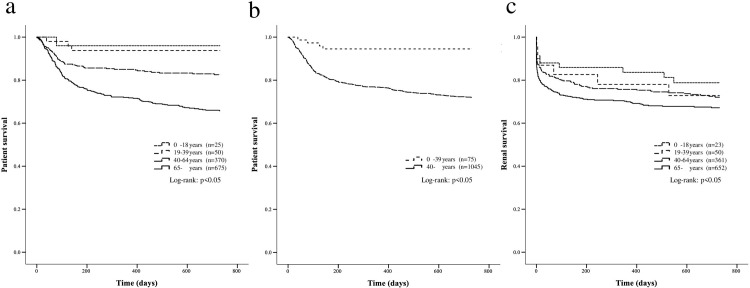
The patient and renal survival in patients with ANCA-associated GN. The comparison of patient survival in patients with ANCA-associated GN among four age groups (A) and two age groups (B), and the renal survival in patients with ANCA-associated GN among four age groups (C) (A, B: n = 1,120, C: n = 1,086).

Among ANCA-associated GN cases, the patient survival rate was 96.0% in children, 94.0% in young adults, 83.8% in the middle-aged, and 70.5% in the elderly, showing the statistically significant differences (p <0.05) (Fig 2A). The patient survival rate of the younger group (children plus young adults) was 94.7%, representing significantly better prognosis compared to 75.2% in the older group (middle-aged plus elderly) (p <0.05) (Fig 2B). The renal survival rate of ANCA-associated GN was 73.9% in children, 80.0% in young adults, 74.2% in the middle-aged, and 70.1% in the elderly, showing a similar tendency to that of RPGN (p = 0.09) (Fig 2C).

Thus, patient survival showed clearly better results in the younger groups both in RPGN and in ANCA-associated GN. The renal survival rate, however, showed a much less pronounced age effect, with only a slight tendency of better prognosis for younger patients and a worse prognosis overall.

## Discussion

What should be noted in this study was that each age group had the characteristics in the percentage within the classification of RPGN and that the younger group (children plus young adults) had a better prognosis for patient survival than the older group (middle-aged and elderly), both in RPGN and in ANCA-associated GN. As for renal survival, there was only a slight tendency (without statistical significance) of a better prognosis in the younger groups (children and young adults) than the older group (middle-aged and elderly). Although the prognoses for RPGN in adults and children were separately reported in many previous reports, studies with a large number of cases focused on age effects have been limited. In this context, age-based prognosis analysis, comparing relatively young patients to older patients, was determined as the main purpose of this study, led by Intractable (former name, Progressive) Renal Diseases Research, Research on intractable disease, from the Ministry of Health, Labour and Welfare of Japan.

Table 4 shows a summary of previous literature in which pediatric rapidly progressive or crescentic glomerulonephritis has been included [7–18]. The patient survival rate was extremely high in these reported cases, consistent with the present study. In contrast to the favorable results for patient survival rate, the renal survival rate was considerably lower; this finding was also seen in the present study. This series of reports suggests that poor renal survival does not necessarily reduce the survival rate in children, contrary to the results of the elderly, who have adverse outcomes for both patient and renal survival. The followings are possible explanations for these results: the difference in proportion of RPGN classification in each age group, the possibility in early detection of the disease in children, the high intensity treatment in children and the vulnerability of the elderly to infection.

**Table 4:** Summary of case series of pediatric RPGN and crescentic glomerulonephritis.

Author	Year of publication	Number (n)	Age at presentation (years)	Male / Female (n)	Clinical features	MPA / GPA (n)	MPO-ANCA / PR3-ANCA (n)	Length of follow-up (years)	Patient survival (n, %)	Renal survival (n, %)
Ellis	1995	5	11.5 ± 2.5	1 / 4	NA	NA	2 / 2	NA	5, 100.0	2, 40.0
Valentini	1998	7	13.0 ± 0.9	2 / 5	NA	3 / 4	3 / 4	4.0 ± 1.0	7, 100.0	6, 85.7
Stegmayr	2000	10	16.5 (median)	5 / 5	RPGN 10	0 / 10	1 / 8	9.0 (median)	10, 100.0	9, 90.0
Hattori	2001	31	11.9 ± 2.9	4 / 27	RPGN 21, ANS 6, CNS 6	21 / 3	28 / 3	3.8 ± 2.2	30, 96.8	22, 71.0
Peco-Antic	2006	7	12.0 ± 2.6	1 / 6	ANS 3, AKI 2	7 / 0	7 / 0	3.0 ± 1.9	7, 100.0	5, 71.4
Siomou	2012	13	13.2 ± 2.9	3 / 10	NA	6 / 7	6 / 7	3.2 ± 2.9	13, 100.0	10, 76.9
Krmar	2013	6	10.6 (median)	1 / 5	AKI 2	NA	6 / 0	4.4 (median)	6, 100.0	6, 100
Noone	2014	40	12.0 (median)	12 / 28	NA	21 / 19	NA	2.4 (median)	40, 100.0	26, 65.0
Khalighi	2015	21	14.0 (median)	6 / 15	NA	NA	10 / 7	2.5 (median)	21, 100.0	14, 66.7
Kouri	2017	22	13.7 (median)	9 / 13	NA	NA	1 / 0	5.8 (median)	22, 100.0	15, 68.2
Piyaphanee	2017	67	10.6 ± 3.0	32 / 35	RPGN 67	NA	NA	1.1 (median)	67, 100.0	43, 64.2
Rianthavorn	2018	72	12.4 (median)	28 / 44	RPGN 38, ANS 30	NA	NA	8.3 (median)	72, 100.0	54, 75.0
Present study		82	11.0 ± 4.8	38 / 44	RPGN 82	5 / 4	22 / 4	2.0	77, 93.9	56, 73.7

NA: not available, RPGN: rapidly progressive glomerulonephritis, ANS: acute nephritic syndrome, AKI: acute kidney injury, CNS: chronic nephritic syndrome.

First, the proportion in RPGN classification was different in each age group, as appeared in the percentage of ANCA-GN in each age group, with 30.5% in children whereas 73.2% in elderly. It could result in the different prognosis, especially ANCA-GN in the older population might be the cause of the poor prognosis. Second, children had high eGFR and low CRP level compared to other age categories, suggesting that they were detected in the early stage of the disease, enabling them to receive early therapeutic intervention. Third, children have a tolerance for high intensity treatment, whereas the elderly is sometimes less aggressively treated due to their vulnerability. Therapeutic approach in children is usually based on adult cases, since little has been known on their management and so the treatment strategy remained to be established in children cases. MP plus CY, as a key treatment applied to severe manifestations, accounted for most of the cases in children in the previous study. Treatment response with MP and CY was favorable, with remission rate of 84% [10]. In the present study, also, children group showed higher frequency of CY use (44%), compared to the older groups. The elderly, on the contrary, often face a problem of intolerance to intensive treatment, forced to choose less aggressive treatment, as shown in the less frequent use of CY. Incomplete treatment resulted in the increased rate of end-stage kidney disease and death, since the disease itself could cause fatal events when the patients were not treated with adequate immunosuppressive therapy. Finally, the elderly are more vulnerable to various complications. Infection is a representative, especially when they receive intensive immunosuppressive therapy. Infection has been known to be the most common cause of death in RPGN [19]. Intensive treatment increases the risk of infection, sometimes resulting in fatal events. Thus, both infection itself and narrowed treatment options decreased survival rate in the elderly. Also, other complications such as cardiovascular disease, diabetes and cancer are more common in the older population. Children, on the contrary, rarely suffer from coexisting diseases, other than renal disease. These reasons may decrease the survival rate in the elderly compared to that of children.

The limitations in this study included the diversity of respondents including primary care physicians, changes in diagnostic methods, differences in how institutions adopted unified treatment procedures, and finally, advances in treatment in the past 30 years, especially the emergence of rituximab.

The present study offered the whole picture of RPGN based on a nationwide survey, especially highlighting age-based differences. Not only did it confirm the unfavorable prognosis in elderly patients, it also indicated that younger age might itself be a good prognostic factor.

## Supporting information

S1 File List of institutions that provided data for this survey. (DOC)
