# Supplementary material for: Age-dependent survival in rapidly progressive glomerulonephritis: A nationwide questionnaire survey from children to the elderly
Source: PLoS One. 2020 Jul 13;15(7):e0236017. doi: 10.1371/journal.pone.0236017 (PMC7357754; doi:10.1371/journal.pone.0236017)
Supplement: S1 File — (DOC) [file pone.0236017.s001.doc]

**Supplementary material**

**List of institutions that provided data for this survey**

Department of Nephrology, Tsukuba Central Hospital; Department of Nephrology, National Tochigi Hospital; Internal Medicine I, Chiba University Hospital; Internal Medicine I, Hamamatsu Medical University Hospital; Department of Pediatrics, Shinshu University Hospital; Internal Medicine II, School of Medicine, Toho University; Department of Nephrology, Tsuruoka Kyoritsu Hospital; Department of Pediatric, Shimoshizu National Hospital; Department of Pediatrics, Chiba University Hospital; Department of Pediatrics, Hamamatsu Medical University Hospital; Department of Pediatrics, Niigata Prefectural Yoshida Hospital; Department of Pediatrics, School of Medicine, Toho University; Department of Nephrology and Rheumatology, Toyota Memorial Hospital; Kagawa Children’s Hospital; Department of Nephrology, Kawasaki Medical University; Department of Pediatrics, Hamamatsu Red Cross Hospital; Department of Internal Medicine, Niigata Prefectural Central Hospital; Department of Nephrology, Toho University Omori Hospital; Nephrology and Dialysis Unit, Misato Kenwa Hospital and Clinic; Department of Pediatric, National Mie Hospital; Department of Pediatrics, Kawasaki Medical University; Department of Nephrology, Hamamatsu Rosai Hospital; Department of Pediatrics, Niigata City Hospital; Blood Purification Center, Tohoku University Hospital; Internal Medicine I, Aichi Medical University Hospital; Department of Pediatric, National Niigata Hospital; Department of Pediatrics, Kawasaki Kyodo Hospital; Internal Medicine II, Toyama Medical University Hospital; Blood Purification Center, Niigata University Medical and Dental Hospital; Department of Internal Medicine, Fujita Health University; Department of Pediatrics, Aichi Medical University Hospital; Department of Pediatric, National Nishisapporo Hospital; Department of Pediatrics, Kawasaki City Hospital; Department of Pediatrics, Toyama Medical University Hospital; Department of Nephrology, Niigata University Hospital; Department of Pediatrics, Fujita Health University; Pediatric Department, Ehime University Medical School Hospital; Department of Pediatric, National Nishitaga Hospital; Department of Pediatrics, Kurashiki Central Hospital; Department of Internal Medicine, Toyama Prefectural Central Hospital; Department of Internal Medicine, Niigata Minami Hospital; Department of Internal Medicine, Tokushima Prefectural Central University; Internal Medicine I, Asahikawa Medical College Hospital; Department of Pediatric, National Chiba-Higashi Hospital; Department of Internal Medicine, Sagamihara Kyodo Hospital; Internal Medicine, Toyama Red Cross Hospital; Department of Pediatric Nephrology, Nippon Steel Yawata Memorial Hospital; Department of Pediatrics, Tokushima University Hospital; Department of Pediatric, Asahikawa Medical College Hospital; Department of Pediatric, National Chubu Hospital; Department of Nephrology, Inoue Hospital; Department of Internal Medicine, Toyama Prefectural Central Hospital; Department of Internal Medicine, Shinnittetsu Hachiman Memorial Hospital; Department of Internal Medicine, Tochigi Saiseikai Utsunomiya Hospital; Department of Nephrology, Anjo Kose Hospital; Department of Nephrology, Kurobe City Hospital; Internal Medicine I, Osaka Medical University Hospital; Department of Pediatrics, Fukui Medical University Hospital; Kobe University School of Medicine, Faculty of Health Sciences; Internal Medicine II, Nara Medical University Hospital; Department of Urology, Kyorin University School of Medicine; Department of Internal Medicine, Saga University; Department of Pediatrics, Osaka Medical University Hospital; Department of Internal Medicine, Fukui Red Cross Hospital; Department of Nephrology, Kanagawa Prefectural Children’s Medical Center; Department of Pediatrics, Nara Medical University Hospital; Internal Medicine I, Kyorin University School of Medicine; Department of Pediatric, Saga University; Internal Medicine I, Osaka City University Hospital; Department of Nephrology, Fukui Red Cross Hospital; Department of Nephrology, Kandatsu Hospital; Department of Nephrology, Minami Ichijo Hospital; Department of Pediatric, Kyorin University School of Medicine; Department of Internal Medicine, Kosei-kan, Saga Prefectural Hospital; Internal Medicine II, Osaka City University Hospital; Internal Medicine IV, Fukuoka University Hospital; Department of Nephrology, Mito Saiseikai General Hospital; Department of Nephrology, Nikko Memorial Hospital; Department of Clinical Genetics, Faculty of Health Sciences, School of Medicine, Kyorin University; Department of Internal Medicine, Saku Sogo Hospital; Department of Pediatrics, Osaka City University Hospital; Internal Medicine I, Fukuoka University Hospital; Department of Internal Medicine, Mito Central Hospital; Department of Nephrology, Hidaka Hospital; Department of Pediatric, Isezaki Municipal Hospital; Department of Internal Medicine, Sano Kosei Sogo Hospital; Osaka Red Cross Hospital; Department of Nephrology, Fukuoka University Hospital; Department of Nephrology, Mizushima Kyodo Hospital; Department of Pediatrics, Red Cross Medical Center; Department of Pediatric, Ibaraki Children’s Hospital; Department of Internal Medicine, Saiseikai Yokohama-shi Nambu Hospital; Internal Medicine I, Osaka University Hospital; Department of Pediatrics, Fukuoka University, Tsukushi Hospital; Internal Medicine I, St. Marianna School of Medicine Hospital; Internal Medicine I, Nippon Medical School Hospital; Department of Nephrology, Ibraki Prefectural Central Hospital; Department of Nephrology, Saiseikai Shimonoseki Sogo Hospital; Department of Pediatrics, Osaka University Hospital; Internal Medicine IV, Fukushima Prefectural Medical University Hospital; Yokohama Seibu Hospital, St. Marianna School of Medicine; Internal Medicine II, Nippon Medical School Hospital; Department of Nephrology, Ibaraki Seinan Medical Center Hospital; Department of Pediatrics, Saiseikai Kurihashi Hospital; NTT West Osaka Hospital; Department of Pediatrics, Fukushima Prefectural Medical University Hospital; Department of Pediatrics, St. Marianna School of Medicine Hospital; Department of Pediatrics, Nippon Medical School Hospital; Department of Nephrology, Utsunomiya Social insurance Hospital; Department of Nephrology, Saiseikai Nakatsu Hospital; Department of Nephrology, Osaka Prefectural Hospital; Internal Medicine V, The Hospital of Hyogo College of Medicine; Department of Pediatrics, Seirei Hamamatsu Hospital; Internal Medicine II, Nippon Medical School Hospital; Department of Internal Medicine, Urasoe Sogo Hospital; Department of Nephrology, Saitama Medical University Hospital; Department of Nephrology, Kumamoto Chuo Hospital; Department of Pediatrics, The Hospital of Hyogo College of Medicine; Department of Pediatrics, St. Lukes International Hospital; Department of Pediatrics, Nippon Medical School Chiba Hokusoh Hospital; Department of Internal Medicine, Yokosuka Kyosai Hospital; Department of Pediatric, Saitama Medical University; Department of Internal Medicine, Oita Medical University Hospital; Department of Pediatric Nephrology, The Hospital of Hyogo College of Medicine; Department of Pediatrics, Seirei Sakura Citizen Hospital; Department of Nephrology, Nihon Red Cross Medical Center; Department of Internal Medicine, Yokosuka City Hospital; Department of Internal Medicine IV, Saitama Medical Center, Saitama Medical University; Department of Urology, Oita Medical University; Hospital Hyogo Prefectural Children’s Hospital; Department of Internal Medicine, Seirei Sakura Citizen Hospital; Internal Medicine II, Nihon University Hospital; Internal Medicine II, Yokohama City University Hospital; Department of Nephrology, Saitama Children’s Medical Center; Department of Pediatrics, Oita Medical University Hospital; Department of Internal Medicine, Hyogo Prefectural Amagasaki Hospital; Department of Urinology, Seirei Sakura Citizen Hospital; Department of Pediatrics, Nihon University Surugadai Hospital; Department of Pediatric, Yokohama City University Medical Center; Department of Internal Medicine II, Sapporo Medical University Hospital; Department of Pediatrics, Yamato City Hospital; Department of Internal Medicine, Toyohashi City Hospital; Department of Internal Medicine, National Cardiovascular Center; Department of Nephrology, Mito General Hospital; Department of Urology, Yokohama Minami Kyosai Hospital; Department of Pediatric, Sapporo Medical University Hospital, Takeshita Hospital; Internal Medicine II, National Defense Medical College; National Health Center for Children’s Health and Development; Department of Nephrology, Hitachi General Hospital; Internal Medicine III, Okayama University Hospital; Department of Internal Medicine, Mitsui Memorial Hospital; Department of Nephrology, Tsukuba Gakuen Hospital; Department of Nephrology, Hokkaido Kinrosha Iryo Kyokai Chuo Hospital; Department of Internal Medicine, Sendai Red Cross Hospital; Department of Pediatrics, Hakodate Goryokaku Hospital; Department of Pediatrics, Okayama University Hospital; Department of Pediatrics, Mitsui Memorial Hospital; Department of Metabolism, Nakadori Sogo Hospital; Department of Pediatrics, Hokkaido University Hospital; Department of Nephrology, Senboku Kumiai Hospital; Department of Nephrology, Hashiro General Hospital; Department of Pediatrics, Okinawa Prefectural Chubu Hospital; Department of Internal Medicine I, Mie University Hospital; Department of Nephrology, Nakagami Hospital; Internal Medicine II, Hokkaido University Hospital; Department of Internal Medicine, Nishi Clinic; Internal Medicine II, Kochi Medical School; Department of Nephrology, Okinawa Prefectural Chubu Hospital; Department of Pediatric, Mie University Hospital; Department of Internal Medicine, Chubu Rosai Hospital; Department of Internal Medicine, Kitamatsu Central Hospital; Department of Nephrology, Nishikobe Medical Center; Department of Pediatrics, Kochi Medical School; Department of Internal Medicine, Okinawa Prefectural Chubu Hospital; Department of Internal Medicine, Saiseikan, Yamagata City Hospital; Department of Internal Medicine, Nakano Sogo Hospital; Department of Nephrology, Hokushin Sogo Hospital; Department of Nephrology, Shizuoka Children’s Hospital; Department of Nephrology, Asahi Chuo Hospital; Internal Medicine II, Kansai Medical University; Department of Urology, Yamagata University Hospital; Internal Medicine II, Nagasaki University Hospital; Department of Nephrology, Kitazato University Hospital; Department of Nephrology, Shizuoka Saiseikai General Hospital; Department of Nephrology, Kasumigaura Medical Center; Department of Pediatrics, Kansai Medical University; Department of Pediatric, Yamaguchi University Hospital; Internal Medicine II, Nagasaki University Hospital; Department of Pediatrics, Kitazato University Hospital; Department of Nephrology, Shizuoka City Hospital; Department of Bacteriology, National Institute of Infectious Diseases; Department of Nephrology, Kansai Rosai Hospital; Department of Nephrology, Yamamoto Kumiai Sogo Hospital; Department of Pediatrics, Nagasaki University Hospital; Department of Nephrology, Hokuriku Central Hospital; Department of Nephrology, Sendai Shakai Hoken Hospital; Department of Internal Medicine I, Kanazawa Hospital; Kidney Dialysis Center, Kanto Hospital; Department of Pediatrics, Yamanashi Medical University Hospital; Department of Internal Medicine, Nagano Red Cross Hospital; Department of Nephrology, Horinouchi Hospital; Department of Nephrology, Chiba Children’s Hospital; Department of Nephrology, Kure Medical Center; Department of Pediatrics, Iwate Medical University; Cardiovascular Internal Medicine, Yamanashi Prefectural Central Hospital; Department of Internal Medicine II, Tottori University Hospital; Department of Nephrology, Honjo Daiichi Hospital; Department of Pediatrics, Medical Center East, Tokyo Women’s Medical University; Department of Nephrology, Takasaki Hospital; Department of Pediatrics, Iwate Prefectural Central Hospital; Department of Internal Medicine, Yamanashi Red Cross Hospital; Department of Pediatrics, Tottori University Hospital; Department of Nephrology, Iizuka Hospital; Department of Internal Medicine, Tokyo Sembai Hospital; Department of Pediatrics, International Medical Center of Japan; Department of Pediatrics, Gifu University Hospital; Department of Nephrology, University of Occupational and Environmental Health, Japan Hospital; Department of Nephrology, Teikyo University Hospital; Department of Pediatrics, Mino City Hospital; Department of Nephrology, School of Medicine and Faculty of Medicine, The University of Tokyo; Department of Nephrology, Department of Pediatrics Internal Medicine III, Kurume University Hospital; Department of Pediatric, University of Occupational and Environmental Health, Japan Hospital; Department of Pediatrics, Teikyo University Hospital; Department of Preventive Medicine, Nagoya University Hospital; Internal Medicine II, School of Medicine and Faculty of Medicine, The University of Tokyo; Internal Medicine II, Hirosaki University Hospital; Department of Pediatrics, Kurume University Hospital; Department of Internal Medicine I, University of Occupational and Environmental Health, Japan Hospital; Department of Internal Medicine III, Teikyo University Hospital; Internal Medicine III, Nagoya University Hospital; Department of Pediatrics, School of Medicine and Faculty of Medicine, The University of Tokyo; Department of Pediatrics, Hirosaki University Hospital; Internal Medicine I, Miyazaki Medical University Hospital; Department of Internal Medicine II, University of Occupational and Environmental Health, Japan Hospital; Department of Pediatrics, Tenri Yorozu Sodanjo Hospital; Department of Internal Medicine, Nagoya University Daiko Medical Center; Department of Urology, Tokyo University Branch Hospital; Department of Pediatrics, Faculty of Medicine, Kagawa University; Department of Pediatrics, Miyazaki Medical University Hospital; Department of Nephrology, Sapporo City Hospital; Department of Nephrology, Tenri Yorozu Sodanjo Hospital; Department of Pediatrics, Nagoya Daiichi Red Cross Hospital; Department of Nephrology, Tokyo Teishin Hospital; Department of Internal Medicine, Faculty of Medicine, Kagawa University; Department of Pediatrics, Kyoto City Hospital; Department of Internal Medicine IV, Akita City Dogo Hospital; Department of Nephrology, Tokyo Metropolitan Cancer and Infectious Diseases Center, Komagome Hospital; Department of Nephrology, Nagoya Daiichi Red Cross Hospital; Department of Nephrology, Tokyo Saiseikai Chuo Hospital; Department of Nephrology, Kagawa Prefectural Chuo Hospital; Cardiovascular Science and Medicine, School of Medicine, Kyoto University; Department of Pediatric, Itoigawa Sogo Hospital; Department of Nephrology Tokyo Metropolitan Kiyose Children’s Hospital; Department of Nephrology, Nagoya Daini Red Cross Hospital; Department of Nephrology, Tokyo Fuchu Hospital; Department of Internal Medicine, Takaoka City Hospital; Department of Pediatrics, Onomichi City Hospital; Department of Internal Medicine, Teraoka Memorial Hospital; Department of Pediatrics, Metropolitan Bokutoh Hospital; Department of Pediatrics, Nagoya Daini Red Cross Hospital; Department of Internal Medicine, Tokyo Rosai Hospital; Department of Urology, Takamatsu City Hospital; Department of Pediatrics, School of Medicine, Kyoto University; Department of Internal Medicine III, Shiga University of Medical Science; Department of Internal Medicine, Metropolitan Bokutoh Hospital; Department of Pediatrics, Tachikawa Sogo Hospital; Department of Nephrology, Tokyo Medical University Hachioji Medical Center; Department of Pediatrics, Showa University Hospital; Kyoto University Health Service; Department of Pediatric, Shiga University of Medical Science; Department of Internal Medicine IV, Shimane Medical University; Internal Medicine III, Ryukyu University Hospital; Department of Nephrology, Tokyo Police Hospital; Department of Internal Medicine, Showa University Fujigaoka Hospital; Internal Medicine II, University Hospital, Kyoto Prefectural University of Medicine; Department of Nephrology, Jichi Medical University; Department of Pediatrics, Shimane Medical University Hospital Department of Pediatrics, Ryukyu University Hospital; Internal Medicine II, The Jikei University; Department of Nephrology, Showa University Hospital; Department of Pediatrics, University Hospital, Kyoto Prefectural University of Medicine; Department of Pediatric, Jichi Medical University; Department of Nephrology, Shimada Memorial Hospital; Internal Medicine III, Wakayama Prefectural Medical University Hospital; Department of Pediatrics, The Jikei University; Department of Nephrology, Kamitsuga General Hospital; Internal Medicine III, Kinki University Hospital; Department of Nephrology, Saitama Medical Center, Jichi Medical University; Department of Nephrology, Tokai University Hospital; Department of Pediatrics, Wakayama Prefectural Medical University Hospital; Department of General Medicine, The Jikei University Kashiwa Hospital; Shinrakuen Hospital; Department of Pediatrics, Kinki University Hospital; Department of Pediatric, Kagoshima City Hospital; Department of Pediatrics, Tokai University Hospital; Department of Nephrology, Komatsu City Hospital; Internal Medicine IV, Tokyo Women’s Medical University Hospital; Department of Pediatrics, Juntendo University Hospital; Department of Nephrology, Kanazawa Medical University; Department of Internal Medicine II, Kagoshima University Hospital; Department of Internal Medicine VII, Tokai University Oiso Hospital; Department of Pediatrics I, Dokyo Medical University; Hospital Department of Urology Kidney Center, Tokyo Women’s Medical University; Department of Internal Medicine III, Gumma University Hospital; Department of Pediatrics, Kanazawa Medical University; Department of Pediatrics, Shakaihoken Chukyo Hospital; Department of Internal Medicine II, Tokyo Medical and Dental University; Department of Nephrology, Tsukuba University Hospital; Department of Internal Medicine, School of Medicine, Keio University; Department of Nephrology, Nihon Red Cross Medical Center; Internal Medicine I, Kanazawa University Hospital; Toride Kyodo Hospital; Department of Nephrology, Tokyo Medical University; Internal Medicine III, Hiroshima Red Cross Hospital &Atomic-Bomb Survivors Hospital; Department of Pediatrics, School of Medicine, Keio University; Department of Nephrology, Juntendo University Hospital; Department of Pediatrics, Kanazawa University Hospital; Department of Nephrology, Akita Kumiai Sogo Hospital; Department of Pediatrics, Tokyo Medical University; Internal Medicine II, Hiroshima University Hospital; Department of Pediatrics, Gunma University Hospital; Department of Pediatrics, Kumamoto Central Hospital; Department of Blood Purification Therapy, Medical School of Kanazawa University; Department of Internal Medicine III, Akita University Hospital; Department of Nephrology, Tokyo Medical University; Kasumigaura Hospital; Department of Pediatrics, Hiroshima University Hospital; Department of Nephrology, Toranomon Hospital; Department of Pediatrics, Onomichi City Hospital; Internal Medicine II, Kyushu University Hospital; Department of Pediatrics, Akita University Hospital; Department of Nephrology, Kensei Sogo Hospital; Department of Nephrology, Showa University Fujigaoka Hospital; Department of Pediatrics, Toranomon Hospital; Department of Pediatrics, Kyushu University Hospital; Department of Nephrology, Akita Rosai Hospital; Department of Nephrology, Hara Urological Clinic; Department of Nephrology, Matsuyama Red Cross Hospital; Department of Pathology I, Shinshu University School of Medicine; Internal Medicine III, Kumamoto University Hospital; Department of Pediatrics, Sumitomo Hospital; Department of Nephrology, Koga Hospital; Department of Pediatrics, Matsuyama Red Cross Hospital; Department of Nephrology, Shizuoka City Hospital; Department of Pediatrics, Kumamoto University Hospital; Department of Pediatrics, Shigei Medical Research Center Hospital; Department of Nephrology, Showa Hospital; Internal Medicine II, Shinshu University Hospital; Department of Nephrology, Sendai Shakai Hoken Hospital; Department of Pediatric Nephrology, Osaka Medical Center and Research Institute for Maternal and Child Health.
